# Gram-negative bacteria activate cellular pathways in plaque microenvironment; Systems biology approach

**DOI:** 10.1186/s12866-025-03933-5

**Published:** 2025-04-24

**Authors:** Reza Ganjali, Mohammad Elahimanesh, Hamidreza Aghazadeh, Mohammad Najafi

**Affiliations:** 1 Clinical Biochemistry Department, Faculty of Medical Sciences, Iran University of Medical Sciences, Tehran, Iran; 2 Islamic Azad University, Tehran Medical Branch, Tehran, Iran; 3 Microbial Biotechnology Research Center, Iran University of Medical Sciences, Tehran, Iran

**Keywords:** Pathway, Porphyromonas gingivalis, Chlamydia pneumoniae, Network, Plaque, Vessel microenvironment

## Abstract

**Background:**

Inflammatory events followed by bacterial infections are related to the progression of the atherosclerosis process. The study investigated the signaling and metabolic pathways of endothelial cells (ECs), macrophages (MQs), vascular smooth muscle cells (VSMCs), and dendritic cells (DCs) after exposure to Gram-negative bacterial infections. Moreover, it aimed at cross-talking and enriching the pathways on the cellular and plaque networks.

**Methods and Materials:**

High-throughput expression data series (*n* = 9) were selected through GEO and MAT data repositories. Upregulated differential expression genes (DEGs) were determined using R software and applied to identify the cellular signaling pathways using Enricher/Reactome tools. Then, the cell networks were visualized using the Cytoscape software and enriched by the pathways of secretory proteins identified using Gene ontology (GO).

**Results:**

The important pathways of the Cytokines (Degree 4, *p* < 6 × 10^–26^), and INF (Degree 4, *p* < 8.6 × 10^–31^) in ECs, Cytokines (Degree 4, *p* < 9.35 × 10^–8^), and GPCR (Degree 3, *p* < 1.45 × 10^–4^) in MQs, NOTCH (Degree 6, *p* < 0.027) in VSMCs, and Cytokines (Degree 4, *p* < 1.45 × 10^–17^) in DCs were found to be activated and enriched after exposure to Gram-negative bacterial infections on the cell networks. Furthermore, the Netrin- 1 (Degree 6, *p* < 0.028), and EGFR (Degree 5, *p* < 0.036) pathways were activated in the intimal thick/xanthoma plaque network while the innate (Degree 9, *p* < 8.9 × 10^–20^) and adaptive (Degree 7, *p* < 4.1 × 10^–12^) immune systems pathways were activated in the fibrous cap atheroma plaque network.

**Conclusion:**

The study revealed the signaling pathways after exposure to Gram-negative bacterial infections on the cell networks in the vessel microenvironment. Furthermore, the cell cross-talks exacerbated these pathways in cells and unstable plaques.

**Clinical Trial Number:**

Not applicable.

**Supplementary Information:**

The online version contains supplementary material available at 10.1186/s12866-025-03933-5.

## Introduction

Cardiovascular diseases are a leading cause of mortality worldwide and pose a significant health concern [1]. The atherosclerosis process progresses through inflammatory events, remodeling of the extracellular matrix, and lipid accumulation in vessel sub-endothelial space, which can lead to plaque rupture and thrombosis [2]. While the modified cholesterol-rich lipoprotein particles contribute to forming the primary core of atherosclerotic plaque, activation of signaling pathways relates to inflammation and cellular dysfunction developing the plaque [3].

Emerging evidence showed that Gram-negative bacterial infections such as *Porphyromonas gingivalis* (*P. gingivalis*) and *Chlamydia pneumonia* (*C. pneumonia*) are involved in the progression of atherosclerosis [4]. Mougeot et al. reported that the most abundant species determined in coronary and femoral vessels is *P. gingivalis* [5]. These bacteria triggered inflammatory pathways and activated the signaling and metabolic axes in the cells [6]. It was reported that the monocytes are polarized to macrophages and dendritic cells through pathogen-associated molecular patterns (PAMP) [7]. These studies proposed that the high levels of Gram-negative bacteria in vessels can initiate a cascade of signaling pathways that leads to inflammation and cellular dysfunction, developing plaque instability and rupture in the vessel microenvironment [8, 9].

Herein, we aimed to explore the interplay linking the endothelial cells (ECs), macrophages (MQs), vascular smooth muscle cells (VSMCs), and dendritic cells (DCs) in response to Gram-negative bacterial infections through an in-depth analysis of high-throughput expression data in the plaque microenvironment. Furthermore, intercellular and intracellular signaling cross-talks were studied in the intimal thick/xanthoma and stable/unstable fibrous cap atheroma plaques. The study also enriched the cellular pathways triggered by Gram-negative bacterial infections on cellular networks.

## Materials and methods

### Data acquisition and screening

Transcriptome data repositories, GEO and Array-express, were screened out for data series that studied the relationships of atherosclerotic plaque and infectious agents using the keywords: “Atherosclerosis”, “Atherosclerotic Plaque”, and “Infection”. Then, the data series were selected based on reports of Gram-negative bacterial infections, microarray data, RNA-Seq data, sample size (n ≥ 2, containing uninfected and infected gene samples), Human, ECs, MQs, VSMCs, and DCs. Other data series related to viral infections and animal experiments were omitted from the study. The plaque data were also limited to intimal thick/xanthoma and stable/unstable fibrous cap atheroma lesions.

### Differential gene expression

R Software (ver. 4.2.2) was utilized to analyze RNA-Seq and microarray datasets. DESeq2 and Limma tools were used to normalize data and identify differentially expressed genes (DEGs). The DEGs with p-values < 0.05 and log2 fold changes (log2 FC) > 2 were determined for further analysis (Supplement 1).

### Cellular pathways

The DEG enrichment was performed using the Enrichr tool (https://maayanlab.cloud/Enrichr). Reactome (signaling/metabolic) pathways were identified in the cells exposed to Gram-negative bacterial infections known as cellular pathways in the ECs, MQs, VSMCs, and DCs. The molecular function and cellular component of secretory proteins obtained from the cellular pathways were determined using the Gene Ontology (GO, https://geneontology.org). The proteins related to cellular receptors were linked with the Reactome pathways.

### Cell and plaque networks

The cell and plaque networks were made by the PathIn online webtool (https://pathin.cing-big.hpcf.cyi.ac.cy) [10] applying pairwise shortest paths option from cellular pathways and were visualized in Cytoscape (https://cytoscape.org). According to the protocol, the edges, known as degrees, were determined by the gene intersections and functional associations between two pathways. Furthermore, the node sizes, known as scores, were estimated from the number of DEGs in the Reactome pathway. The cellular high-degree pathways were reported by adjusted p-values < 0.05 based on the ratio of observed DEGs to total genes in each Reactome pathway (Supplement 2). The intersection nodes between the cellular pathways and the pathways linked with secretory proteins were highlighted on the cell and plaque networks. The sub-clusters with nodes < 4 were removed from the networks.

## Results

### Data series

The study data series and their characteristics are presented in Tables 1 and 2.

**Table 1:** Data series in the study

No	Accession number^1^	Platform	Publication (PMID)
1	GSE24897	GPL570	21,203,416
2	GSE67141	GPL16791	26,466,817
3	GSE19590	GPL9365	NA
4	GSE27008	GPL570	NA
5	GSE12806	GPL570	19,643,200
6	GSE28829	GPL570	22,388,324
7	GSE120521	GPL16791	31,339,449
8	E-MTAB- 1922	NA	24,209,892
9	E-MTAB- 3955	NA	NA

The data series are obtained from GEO/NCBI and ArrayExpress/EMBL

^1^. GSE, Gene Expression Omnibus Series. NA, Not available

**Table 2:** Features of the study data series

Accession number	Data type	Sample^a^	Description^b^
GSE24897	Array	GSM612262, GSM612263, GSM612264, GSM612265, GSM612266, GSM612267, GSM612268, GSM612269, GSM612270, GSM612271, GSM612272, GSM612273	Macrophages uninfected and infected with live Pg, Pg liposaccharide and fimbriae
GSE67141	RNA-Seq	GSM1640152, GSM1640153	Human myeloid dendritic cells uninfected and infected with Pg
GSE19590	Array	GSM460660, GSM460659, GSM460658	Human atheroma plaques uninfected and infected with Cp
GSE27008	Array	GSM665337, GSM665341	Human coronary endothelial cells uninfected and infected with Cp
GSE12806	Array	GSM321605, GSM321606, GSM321607, GSM321608	Dendritic cells uninfected and infected with Cp
GSE28829	Array	GSM714070, GSM714071, GSM714072, GSM714073, GSM714074, GSM714075, GSM714076, GSM714077, GSM714078, GSM714079, GSM714080, GSM714081, GSM714082, GSM714083, GSM714084, GSM714085, GSM714086, GSM714087, GSM714088, GSM714089, GSM714090, GSM714091, GSM714092, GSM714093, GSM714094, GSM714095, GSM714096, GSM714097, GSM714098	Advanced and early atherosclerotic plaques
GSE120521	RNA-Seq	GSM3402504, GSM3402505, GSM3402506, GSM3402507, GSM3402508, GSM3402509, GSM3402510, GSM3402511	Stable and unstable atheroma sections
E-MTAB- 1922	Array	All four samples. Dual channel	Human aortic smooth muscle cells uninfected and infected with Pg
E-MTAB- 3955	Array	Samples 381, 382, 383, w1, w2, w3, ct1, ct2, and ct3	Human aortic smooth muscle cells uninfected and infected with Pg

^a^. GSM, Gene Expression Omnibus Sample

^b^. Cp, *Chlamydia pneumonia*. Pg, *Porphyromonas gingivalis*

### The cells exposed to Gram-negative bacterial infections in plaque microenvironment

#### Endothelial cells (ECs)

**Fig. 1:**
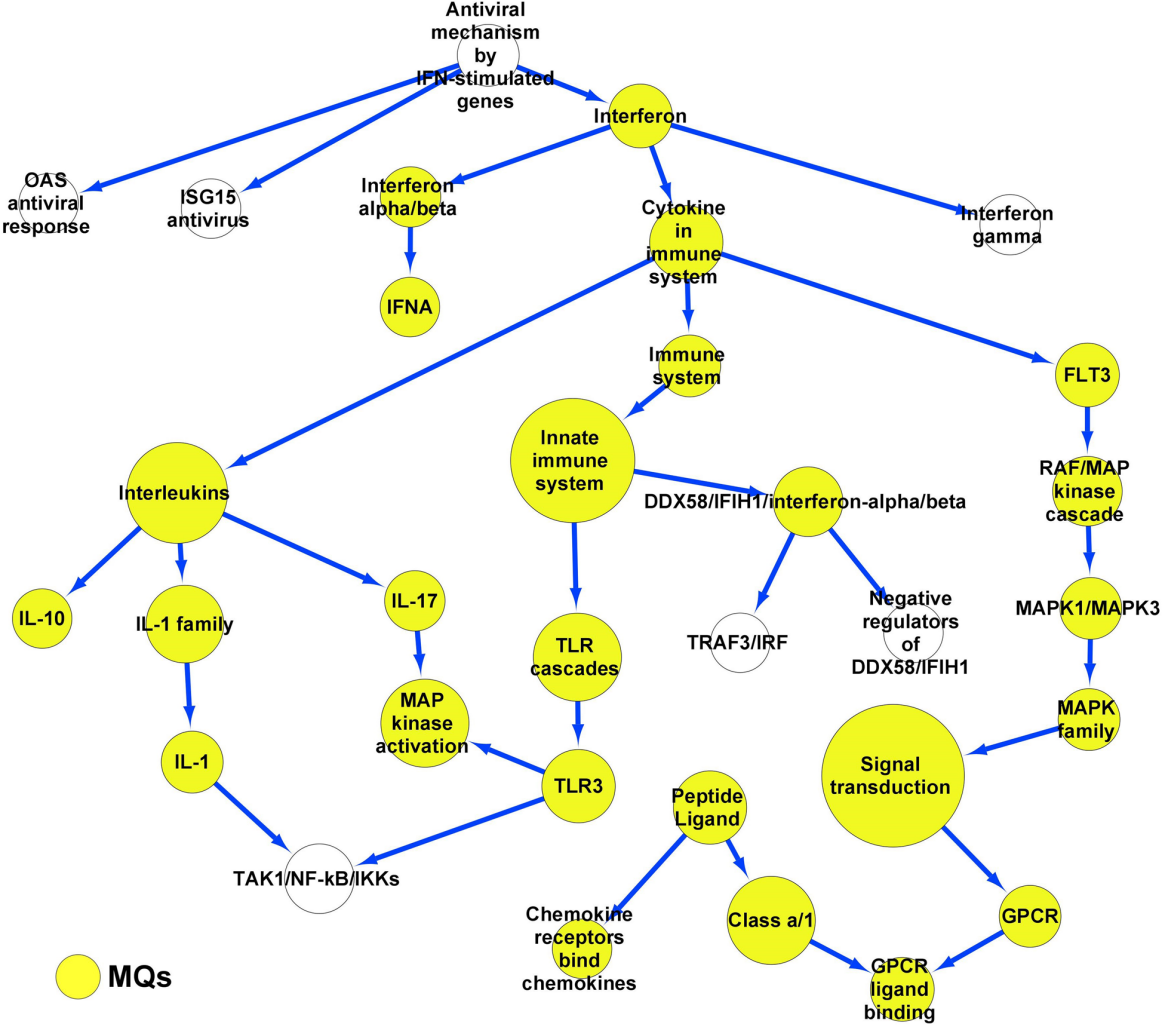
The endothelial cell network. The DEGs were determined from endothelial cells after exposure to Gram-negative bacterial infections. A network was made from the cellular pathways related to the DEGs. The network was also enriched using pathways linked with proteins secreted from macrophages after exposure to Gram-negative bacterial infections. DEGs, Differential expression genes. Macrophages, MQs

72 upregulated DEGs by analyzing the GSE27008 dataset were determined and subjected to the EC pathways (n = 22). Then, a network was made through the EC pathways containing 32 nodes and 33 edges (Fig. 1). The high-degree nodes in the network included the Cytokines (Degree 4, *p* < 6 × 10^–26^), Interleukins (Degree 4, *p* < 0.033), INF (Degree 4, *p* < 8.6 × 10^–31^), Innate immune system (Degree 3, *p* < 0.02), and TLR (Degree 3, *p* < 0.02) signaling pathways as reported in Table 3. It was enriched with the pathways linked with the secretory proteins of MQs (Yellow). The pathways of network included 2,335 proteins, of which 1,110 were found in the extracellular region, and 199 had receptor activator activity. Of these, 196 proteins exhibited both receptor activator activity and extracellular localization and were involved in 74 cellular pathways.

**Table 3:** Cellular high-degree pathways in endothelial cells exposed to Gram-negative bacterial infections

**Hub**^**a**^ (Signaling/Metabolism)	**Degree**^**b**^	**First neighbors**^**c**^ (Signaling /Metabolism)	**Second neighbors**^**d**^ (Signaling /Metabolism)
Cytokine	4	Interleukins, INF, Immune system, FLT3	IL- 10, IL- 1, IL- 17, INF alpha/beta, Innate immune system, Antiviral/IFN
Interleukins	4	IL- 10, IL- 1, IL- 17, Cytokine	IL- 1, MAPK, Interferon, Immune system, FLT3
INF	4	INF alpha/beta, INF gamma, Antiviral/IFN	IFN alpha, ISG15 antiviral mechanism, OAS antiviral response
Innate immune system	3	Immune system, TLR, DDX58/IFIH1/INF alpha/beta	TRAF3/IRF, TLR3, Cytokine
TLR3	3	TAK1/NF-kB/IKKs, MAPK, TLR	IL- 1, IL- 17, Innate immune system
Antiviral/IFN	3	INF, ISG15 antiviral mechanism, OAS antiviral response	INF gamma, INF alpha/beta

^a^. Hub, High-degree signaling/metabolic pathways in the EC network

^b^. Degree, The edges of Hub

^c^. First neighbors, The first level of cellular pathways close to the hub pathway in the EC network

^d^. Second neighbors, The second level of cellular pathways close to the hub pathway in the EC network

Degree > 2, Endothelial cell, EC

#### Macrophages (MQs)

**Fig. 2:**
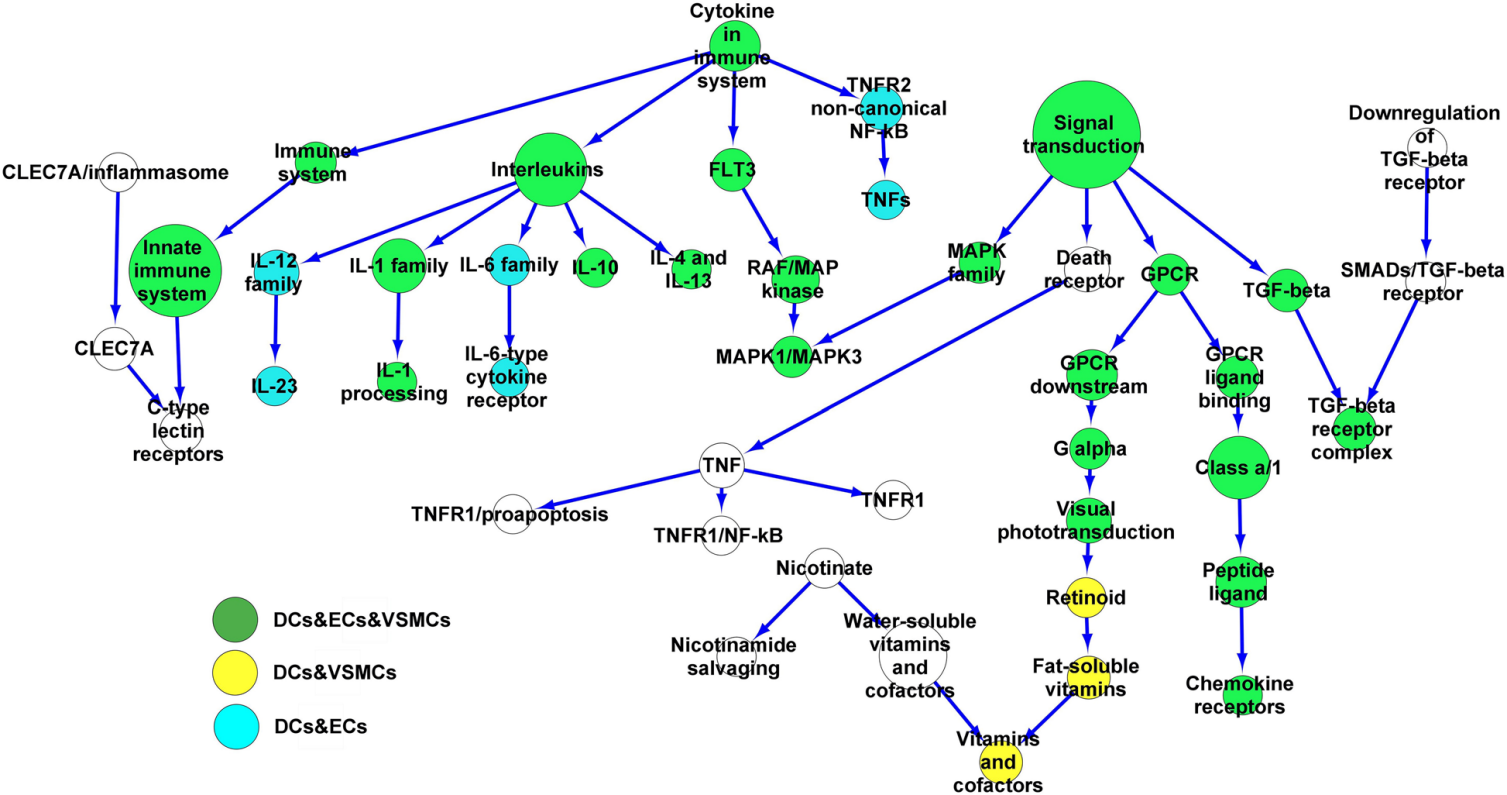
The macrophage network. The DEGs were determined from macrophages exposed to Gram-negative bacterial infections. The network was made from the cellular pathways related to the DEGs. Moreover, the network was enriched using pathways linked with proteins secreted from the activated dendritic cells, vascular smooth muscle cells, and endothelial cells by Gram-negative bacterial infections. DEGs, Differential expression genes. Dendritic cells, DCs. Endothelial cells, ECs. Vascular smooth muscle cells, VSMCs

Analyzing the GSE24897 dataset identified the upregulated DEGs (*n* = 71). The DEGs were found in 34 MQ pathways and used to make a network (containing 45 nodes and 44 edges) (Fig. 2). The high-degree nodes on the network included the Interleukins (Degree 6, p < 0.021), Cytokines (Degree 4, *p* < 9.35 × 10^–8^), and, GPCR (Degree 3, *p* < 1.45 × 10^–4^) signaling pathways as showed in Table 4. The network included 4,233 proteins related to the MQ pathways. 322 proteins were extracellularly localized and had receptor activator activity according to GO data. These secretory proteins were involved in 208 pathways. The MQ network was enriched using the pathways linked with the DC, EC, and VSMC secretory proteins. The node intersections with all of the cells (DCs, ECs, and VSMCs, Green), with both the DCs and VSMCs (Yellow), and with both the DCs and ECs (Blue) are shown on the network (Fig. 2).

**Table 4:** Cellular high-degree pathways in macrophages exposed to Gram-negative bacterial infections

**Hub**^**1**^ (Signaling/Metabolism)	**Degree**^**2**^	**First neighbors**^**3**^ (Signaling/Metabolism)	**Second neighbors**^**4**^ (Signaling/Metabolism)
Interleukins	6	IL- 10, IL- 6, IL- 4 and IL- 13, IL- 1, IL- 12, Cytokine	IL- 6, IL- 1, IL- 23, Immune system, FLT3, TNFR2/NF-kB
Cytokine	4	Immune system, Interleukins, FLT3, TNFR2/NF-kB	Innate immune system, TNFs, RAF/MAPK, IL- 10, IL- 6, IL- 4 and IL- 13, IL- 1, IL- 12
Signal transduction	4	MAPK, TGF-beta, Death receptor, GPCR	TGF-beta, MAPK1/MAPK3, TNF, GPCR, GPCR
TNF	4	Death receptor, TNFR1/proapoptotic, TNFR1/NF-kB, TNFR1	Signal transduction
GPCR	3	Signal transduction, GPCR ligand binding, GPCR	Class a/1, G alpha, TGF-beta, MAPK, Death receptor
Innate immune system	3	Toll-like receptor cascade, C-type lectin receptors, Immune system	CLEC7 A, Cytokine

^1^. Hub, High-degree signaling/metabolic pathways in the MQ network

^2^. Degree, The edges of Hub

^3^. First neighbors, The first level of cellular pathways close to the hub pathway in the MQ network

^4^. Second neighbors, The second level of cellular pathways close to the hub pathway in the MQ network

Degree > 2. Macrophage, MQ

#### Vascular smooth muscle cells (VSMCs)

**Fig. 3:**
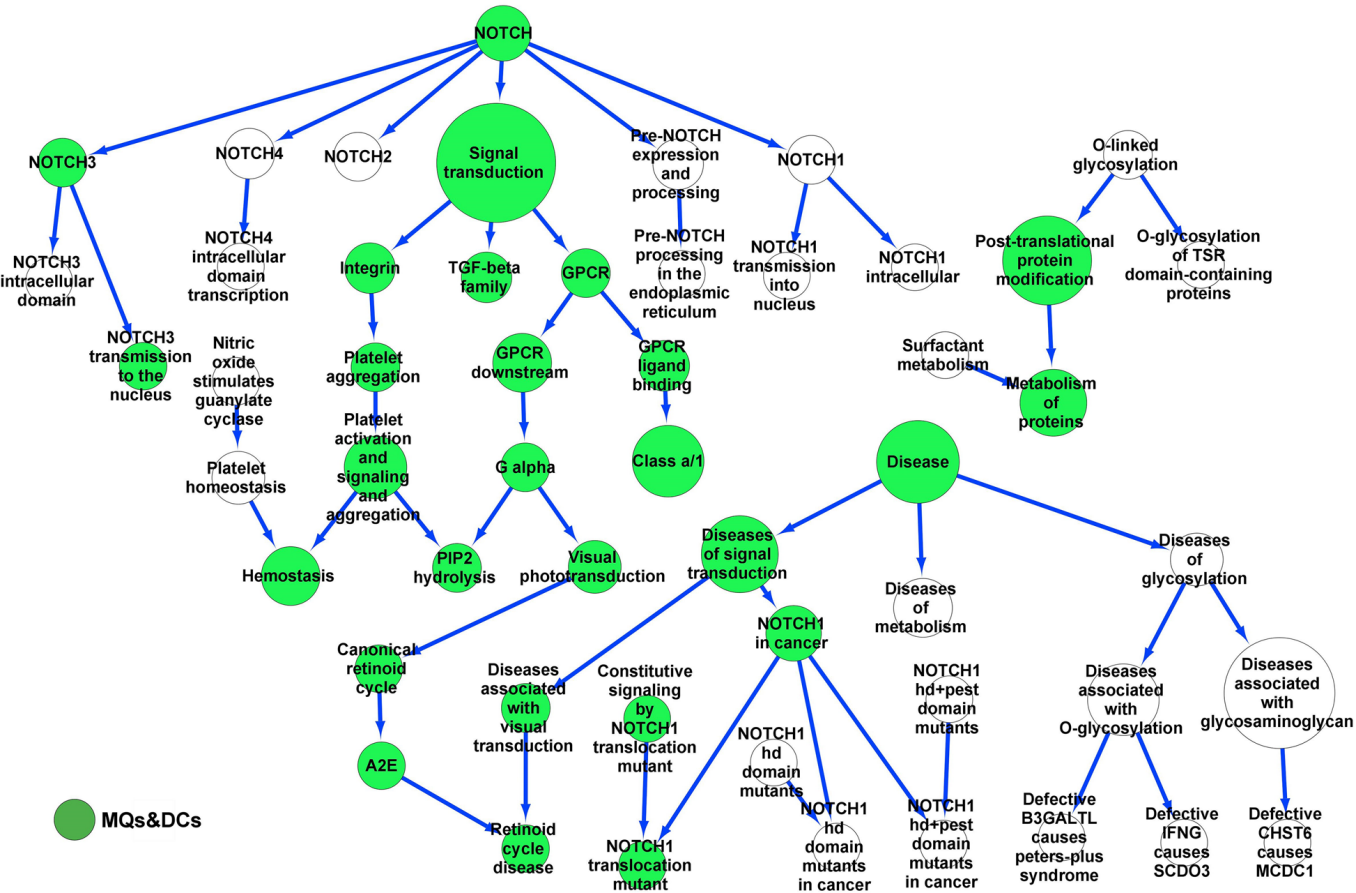
The vascular smooth muscle cell network. The DEGs were found in vascular smooth muscle cells after exposure to Gram-negative bacterial infections. The network was made from the cellular pathways related to the DEGs. Then, it was enriched using pathways linked with proteins secreted from the activated macrophages, and dendritic cells by Gram-negative bacterial infections. DEGs, Differential expression genes. Macrophages, MQs. Dendritic cells, DCs

The DEGs from E-MTAB- 1922 (*n* = 131) and E-MTAB- 3955 (*n* = 76) datasets were found in VSMC pathways (*n* = 37). A network containing 52 nodes and 51 edges was made using the VSMC pathways (Fig. 3). The high-degree nodes included the NOTCH (Degree 6, *p* < 0.027), GPCR (Degree 3, *p* < 0.03), and Diseases of Glycosylation (Degree 3, *p* < 0.0036) pathways as described in Table 5. The VSMC network included 4,348 proteins, of which 1,473 were extracellular, and 271 had receptor activator activity. 257 proteins localized in the extracellular matrix, had receptor activator activity, and were linked with 200 pathways. The VSMC network was enriched with the pathways related to the secretory proteins of MQs and DCs. The node intersections with both DCs and MQs (Green) are highlighted on the network (Fig. 3).

**Table 5:** Cellular high-degree pathways in smooth muscle cells exposed to Gram-negative bacterial infections

**Hub**^**a**^ (Signaling/Metabolism)	**Degreeb**^**2**^	**First neighbors**^**c**^ (Signaling /Metabolism)	**Second neighbors**^**d**^ (Signaling /Metabolism)
NOTCH	6	Signal transduction,, pre-NOTCH, NOTCH2 NOTCH1, NOTCH4, NOTCH3	TGF-beta, Integrin, GPCR, Pre-NOTCH, NOTCH1, NOTCH1 transmission, NOTCH4, NOTCH3 transmission, NOTCH3
Signal transduction	4	TGF-beta, Integrin, NOTCH, GPCR	Platelet aggregation, GPCR, GPCR ligand binding, Pre-NOTCH, NOTCH2, NOTCH4, NOTCH3
NOTCH1	3	NOTCH, NOTCH1, NOTCH1 transmission	Signal transduction, NOTCH4, NOTCH3, NOTCH2, Pre-NOTCH
NOTCH3	3	NOTCH, NOTCH3 transmission, NOTCH3	Signal transduction, NOTCH2, NOTCH1, NOTCH4, NOTCH3, Pre-NOTCH
GPCR	3	Signal transduction, GPCR, GPCR ligand binding	Class A/1, Visual phototransduction, PIP2 hydrolysis, NOTCH, Integrin, TGF-beta
Platelet	3	Platelet aggregation, Hemostasis, PIP2 hydrolysis	Integrin, Platelet hemostasis, GPCR, Visual phototransduction
G alpha	3	GPCR, PIP2 hydrolysis, Visual phototransduction	Retinoid cycle, GPCR, Platelet activation and aggregation
Diseases of signal transduction	3	NOTCH1 in cancer, Disease, Diseases associated with visual transduction	Retinoid cycle disease, Diseases, Diseases of glycosylation, NOTCH1, NOTCH1 mutants in cancer
NOTCH1 in cancer	3	NOTCH1 translocation mutant, NOTCH1 hd mutants in cancer, NOTCH1 hd + pest mutants in cancer, diseases	NOTCH1 translocation mutant, NOTCH1 hd domain mutants, NOTCH1 hd + pest mutants, Disease, Diseases with visual transduction
Disease	3	Diseases of signal transduction, metabolism, glycosylation	Diseases associated with glycosaminoglycan metabolism, Diseases associated with O-glycosylation of proteins, Signaling by NOTCH1 in cancer, Diseases associated with visual transduction
Diseases of glycosylation	3	Disease, diseases associated with glycosaminoglycan, O-glycosylation	CHST6/MCDC1,IFNG/SCDO3, B3GALTL/peter-plus syndrome, Diseases
Diseases of O-glycosylation	3	Diseases of glycosylation, IFNG/SCDO3	Disease, Diseases associated with glycosaminoglycan

^a^. Hub, High-degree signaling/metabolic pathways in the VSMC network

^b^. Degree, The edges of Hub

^c^. First neighbors, The first level of cellular pathways close to the hub pathway in the VSMC network

^d^. Second neighbors, The second level of cellular pathways close to the hub pathway in the VSMC network

Degree > 2. Vascular smooth muscle cell, VSMC

#### Dendritic cells (DCs)

**Fig. 4:**
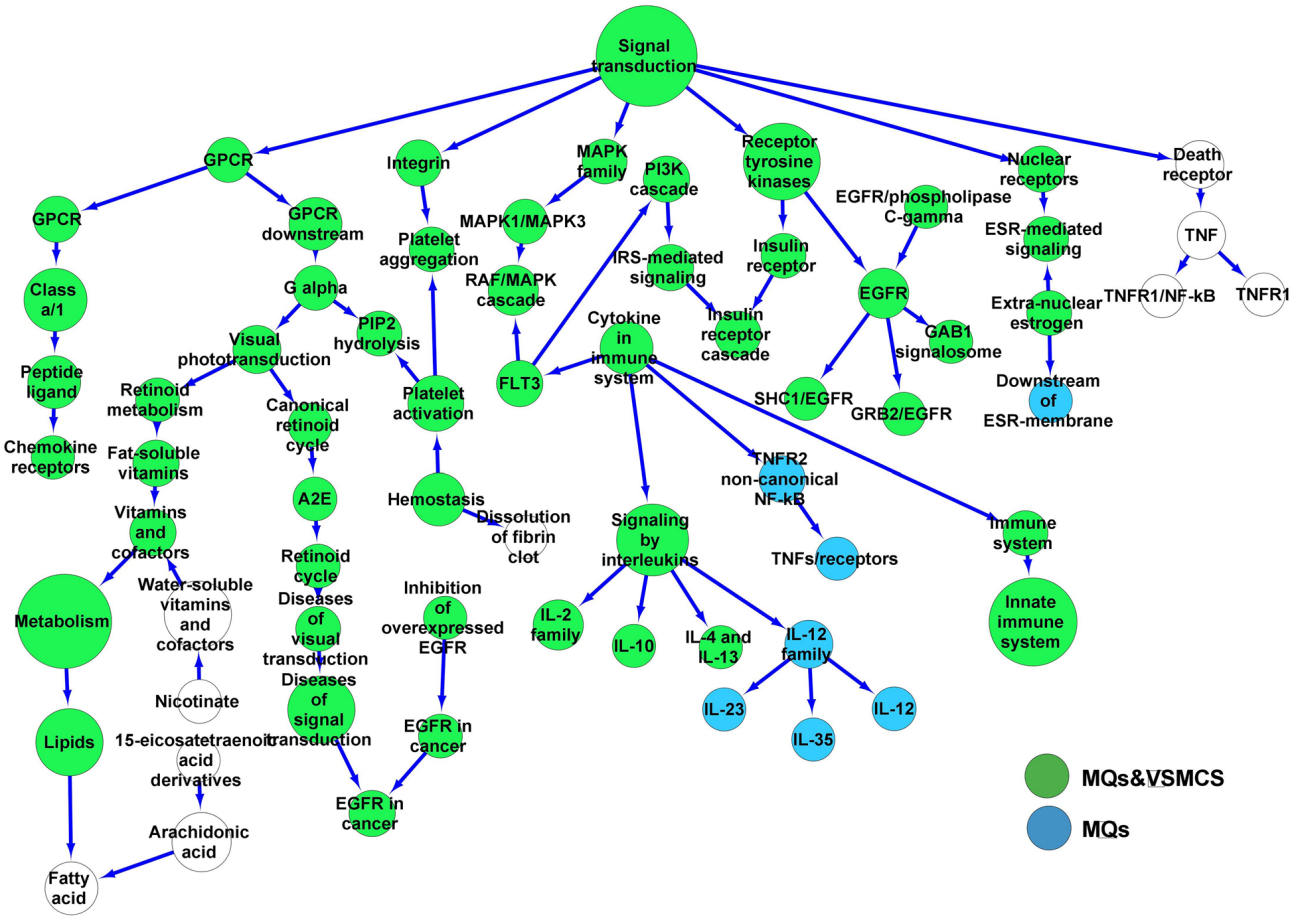
The dendritic cell network. The DEGs were identified from the activated dendritic cells by Gram-negative bacterial infections. The DC network was made from the cellular pathways related to the DEGs. The network was also enriched using pathways linked with proteins secreted from macrophages, and vascular smooth muscle cells exposed to Gram-negative bacterial infections. DEGs, Differential expression genes. Macrophages, MQs. Vascular smooth muscle cells, VSMCs

Eighty one DEGs by analyzing the GSE67141 and GSE12806 data series found in 38 DC pathways and used to make a network containing 68 nodes and 69 edges (Fig. 4). The high-degree nodes included the Interleukins (Degree 5, *p* < 0.011), EGFR (Degree 5, *p* < 0.013), GPCR (Degree 3, *p* < 0.025), and Cytokines (Degree 4, *p* < 1.45 × 10^–17^) signaling pathway as reported in Table 6. The DC network included 4,497 proteins (containing 1,798 extracellular proteins and 341 possessing receptor activator activity). 332 proteins were extracellularly localized and had receptor activator activity. The proteins were linked with 207 pathways. The DC network was enriched with the pathways obtained from MQ and VSMC secretory proteins. The node intersections with MQs (blue), and with both VSMCs and MQs (Green) are shown on the DC network.

**Table 6:** Cellular high-degree pathways in dendritic cells exposed to Gram-negative bacterial infections

**Hub**^**a**^ (Signaling/Metabolism)	**Degree**^**b**^	**First neighbors**^**c**^ (Signaling /Metabolism)	**Second neighbors**^**d**^ (Signaling /Metabolism)
Signal transduction	6	Nuclear receptors, Integrin, GPCR, Receptor tyrosine kinases, MAPK, death receptor	ESR, Platelet activation and aggregation, GPCR, EGFR, Insulin receptor, MAPK1/MAPK3, TNF
Interleukins	5	IL- 10, IL- 12, IL- 2, IL- 4 and IL- 13, Cytokine	IL- 35, IL- 23, IL- 12, FLT3, TNFR2/NF-kB
EGFR	5	SHC1/EGFR, GRB2/EGFR, EGFR/phospholipase C-gamma, GAB1 signalosome, Receptor tyrosine kinases	Insulin receptor
Cytokine	4	Interleukins, TNFR2/NF-kB, FLT3	RAF/MAPk, PI3 K, TNFs, Innate immune system, IL- 10, IL- 12, IL- 2, IL- 4 and IL- 13
IL- 12 family	4	Interleukins, IL- 35, IL- 23, IL- 12	IL- 10, IL- 2, IL- 4, IL- 13, Cytokine
Vitamins and cofactors	3	Metabolism, Fat-soluble vitamins, Water-soluble vitamins, cofactors	Lipids, Nicotinamide, Retinoid
Visual photo transduction	3	G alpha, Retinoid receptors, Retinoid	Fat-soluble vitamins, A2E/Retinal degradation, PIP2 hydrolysis, GPCR
G alpha	3	Visual phototransduction, GPCR, PIP2 hydrolysis	GPCR, Platelet activation and aggregation, Retinoid cycle, Retinoid metabolism
GPCR	3	Signal transduction, GPCR	Class a/1, G alpha, Nuclear receptors, Integrin, receptor tyrosine kinases, MAPK, Death receptor
Receptor tyrosine kinases	3	EGFR, Insulin receptor, Signal transduction	SHC1/EGFR, GRB2/EGFR, EGFR/phospholipase C-gamma, GAB1 signalosome, Insulin receptor, GPCR, Nuclear receptors, Integrin, Death receptor, MAPK
TNF	3	TNFR1, Death receptor, TNFR1/NF-kB	Signal transduction
FLT3	3	Cytokine, RAF/MAP, PI3 K	MAPK1/MAPK3, IRS-mediated, interleukins, Immune system, TNFR2/NF-kB
Platelet	3	Hemostasis, Platelet aggregation, PIP2 hydrolysis	Fibrin clot, G alpha, Integrin

^a^. Hub, High-degree signaling/metabolic pathways in the DC network

^b^. Degree, The number of edge

^c^. First neighbors, The first level of cellular pathways close to the hub pathway in the DC network

^d^. Second neighbors, The second level of cellular pathways close to the hub pathway in the DC network

Degree > 2. Dendritic cell (DC)

### Intimal thick/xanthoma and stable/unstable fibrous cap atheroma plaques

**Fig. 5:**
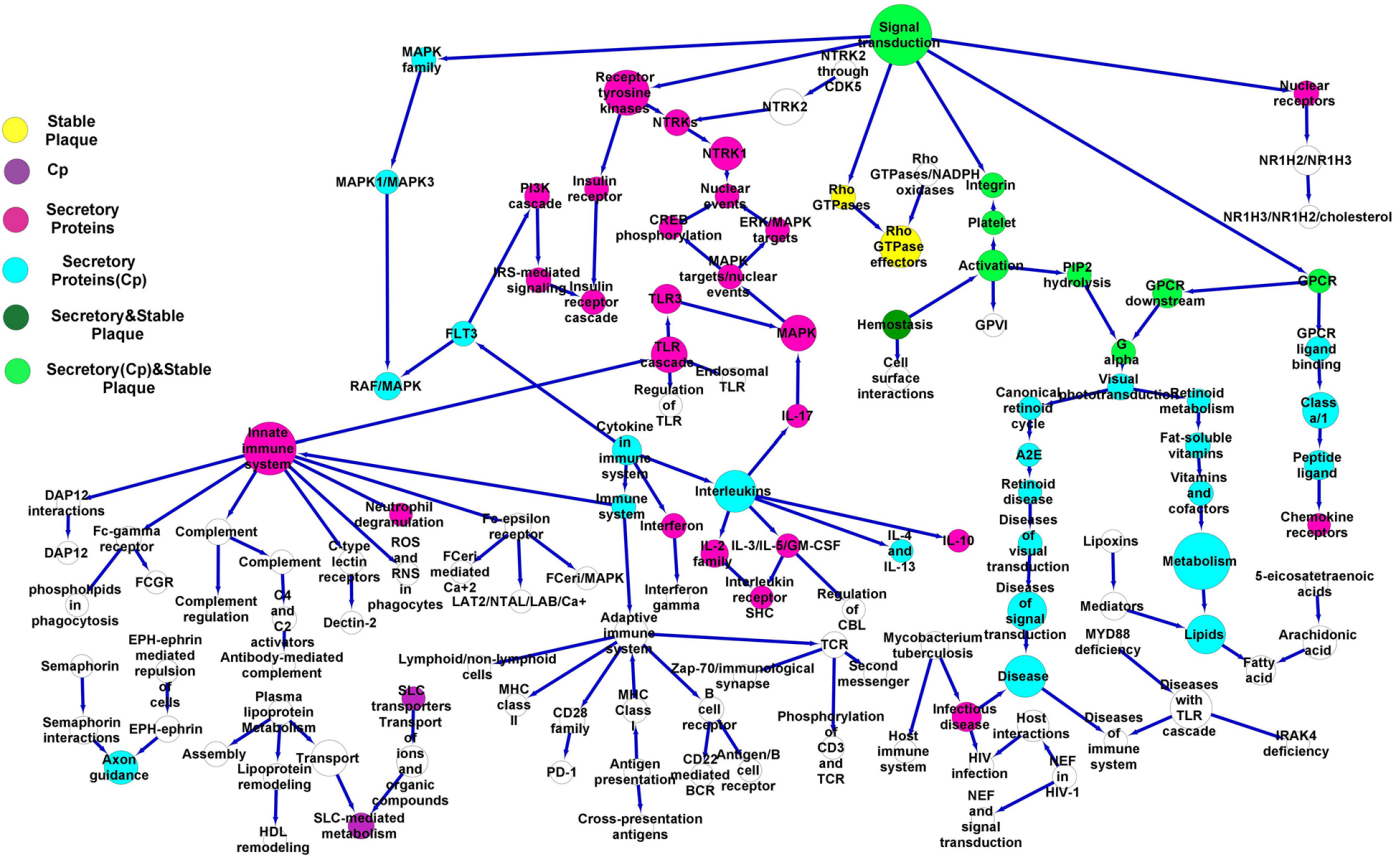
Stable fibrous cap atheroma plaque network. The DEGs were identified from fibrous cap atheroma plaques. The network was made from the cellular pathways related to the DEGs and was enriched using pathways identified from stable plaques. Furthermore, the pathways linked with proteins secreted from macrophages, dendritic cells, vascular smooth muscle cells, and endothelial cells after exposure to Gram-negative bacterial infections were highlighted on the network. DEGs, Differential expression genes. Dendritic cells, DCs. Endothelial cells, ECs. Vascular smooth muscle cells, VSMCs. Macrophages, MQs

**Fig. 6:**
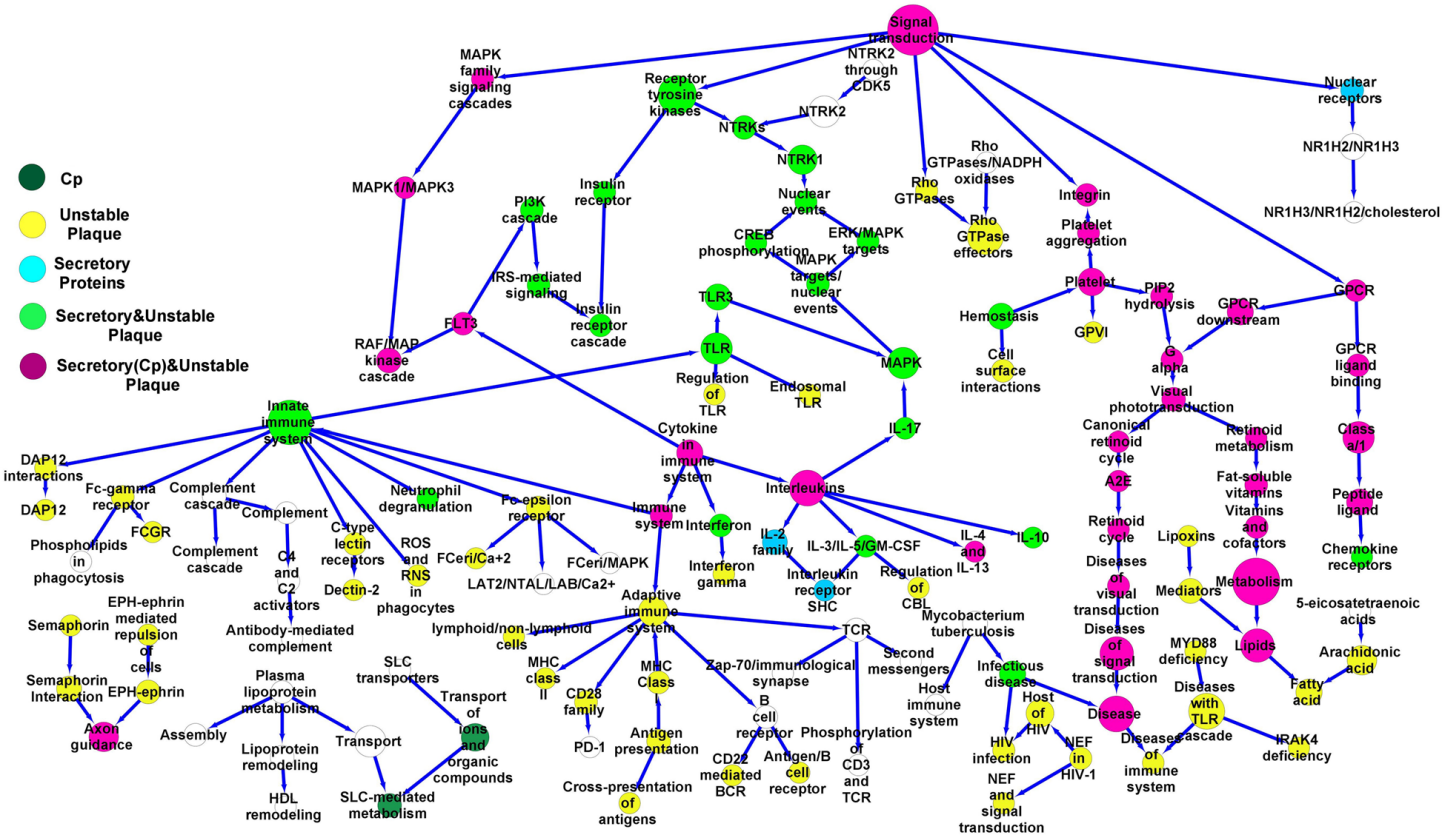
Unstable fibrous cap atheroma plaque network. The DEGs were identified from fibrous cap atheroma plaques. The network was made from the cellular pathways related to the DEGs and was enriched using pathways obtained from unstable plaques. Furthermore, the pathways linked with proteins secreted from the activated macrophages, dendritic cells, vascular smooth muscle cells, and endothelial cells by Gram-negative bacterial infections were identified on the network. DEGs, Differential expression genes. Dendritic cells, DCs. Endothelial cells, ECs. Vascular smooth muscle cells, VSMCs. Macrophages, MQs

**Fig. 7:**
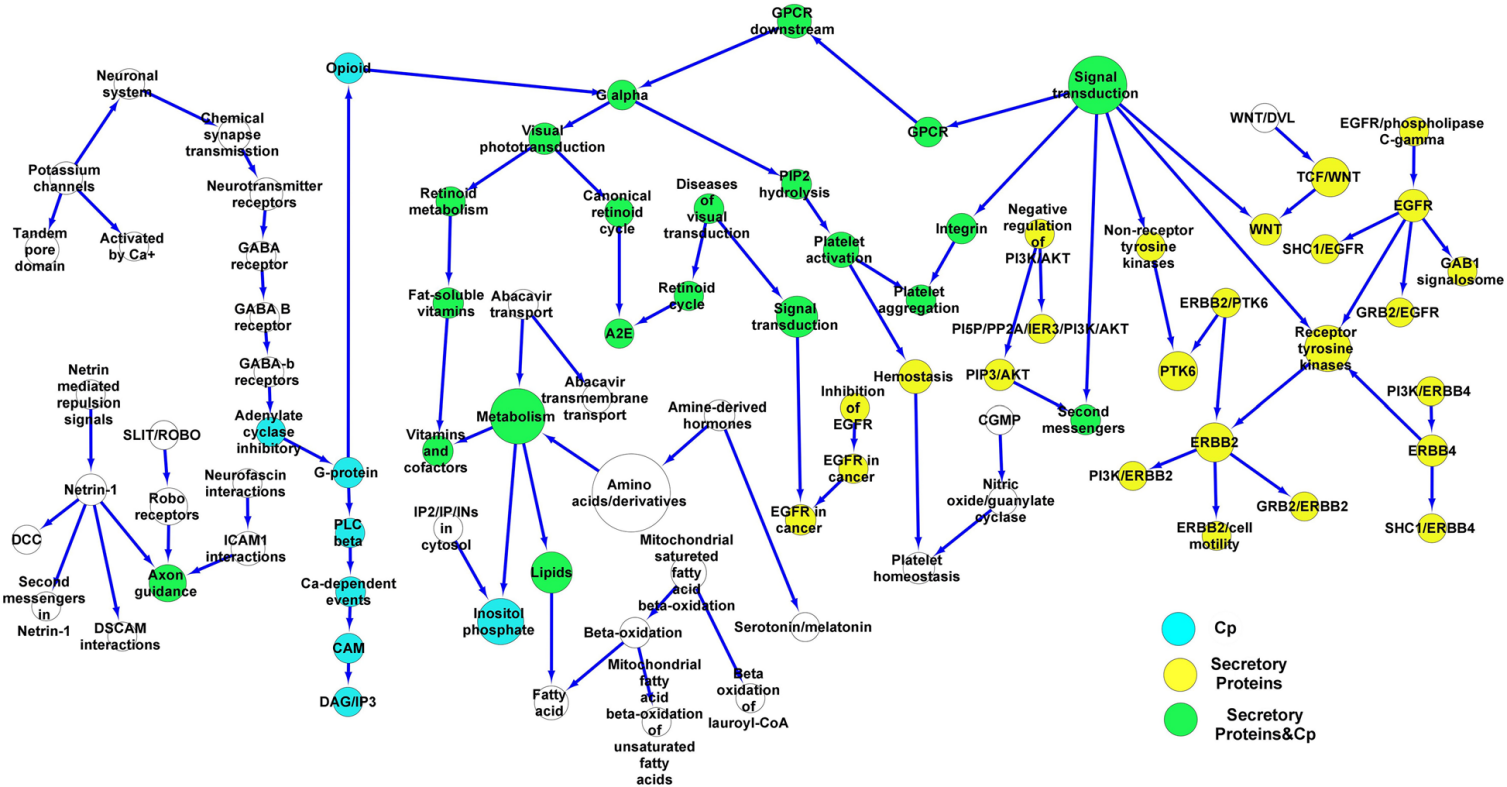
Intimal thick/xanthoma plaque network. The DEGs were found from intimal thick/xanthoma plaques. The network was made from the cellular pathways related to the DEGs. Furthermore, the network was enriched using pathways linked with proteins secreted from macrophages, dendritic cells, vascular smooth muscle cells, and endothelial cells after exposure to Gram-negative bacterial infections. DEGs, Differential expression genes. Dendritic cells, DCs. Endothelial cells, ECs. Vascular smooth muscle cells, VSMCs. Macrophages, MQs

The GSE28829 dataset reported pathological plaques with intimal thick/xanthoma and fibrous cap atheroma lesions. The upregulated DEGs (*n* = 372 and *n* = 65, respectively) were linked with cellular pathways and applied in the fibrous cap atheroma (87 nodes and 88 edges) and intimal thick/xanthoma plaque networks (130 nodes and 132 edges) (Figs. 5, 6, and 7). The high-degree nodes in the fibrous cap atheroma plaque network included innate (Degree 9, *p* < 8.9 × 10^–20^) and adaptive (Degree 7, *p* < 4.1 × 10^–12^) immune systems, Interleukins (Degree 6, *p* < 9.5 × 10^–8^), platelet activation (Degree 4, *p* < 1.45 × 10^–5^), Cytokines (Degree 4, *p* < 1.12 × 10^–9^), and TCR (Degree 4, *p* < 0.012) pathways and, in the intimal thick/xanthoma plaque network included the Netrin- 1 (Degree 6, *p* < 0.028), EGFR (Degree 5, *p* < 0.036), and ERBB2 (Degree 5, *p* < 0.045) pathways as shown in Tables 7 and 8, respectively.

**Table 7:** Cellular high-degree pathways in the fibrous cap atheroma plaques

**Hub**^**a**^ (Signaling/Metabolism)	**Degree**^**b**^	**First neighbors**^**c**^ (Signaling/Metabolism)	**Second neighbors**^**d**^ (Signaling/Metabolism)
Innate immune system	9	Neutrophil degranulation, C-type lectin receptors, Fc-gamma receptor, Fc-epsilon receptor, Complement, DAP12, Immune system, ROS/RNS/Phagocytes, TLR	Adaptive immune system, Cytokine, Dectin- 2, Phospholipids in phagocytosis, FCGR, FCeri/MAPK, LAT2/NTAL/LAB/Ca, FCeri/Ca, Complement, DAP12, TLR, TLR, TLR3
Adaptive immune system	7	MHC Class II antigen, CD28, MHC Class I antigen, TCR, B cell receptor, Immune system, Immunoregulatory Lymphoid/non-lymphoid cells	PD- 1, Antigen presentation, Second messenger molecules, CD3/TCR, Zap70/synapse, Antigen/B cell receptor, CD22/BCR, Innate immune signaling, Cytokine
Interleukins	6	IL- 17, IL- 2, IL- 10, IL- 3 and IL- 5 and GM-CSF, IL- 4 and IL- 13, Cytokine	CBL, Interleukin receptor/SHC, TLR3, MAPK, Immune system, FLT3
Signal transduction	6	Rho GTPases, GPCR, MAPK, Integrin, Nuclear receptors, Receptor tyrosine kinases	Rho GTPase, GPCR, GPCR ligand binding, Platelet aggregation, NR1H2 and NR1H3, MAPK1/MAPK3, Insulin receptor. NTRKs
Platelet	4	Hemostasis, GPVI, Platelet aggregation, PIP2 hydrolysis,	Integrin, Cell adhesion, G alpha
Cytokine	4	Interleukins, immune system, FLT3, INF	INF gamma, Adaptive immune system, Innate immune system, RAF/MAPK, PI3 K, IL- 17, IL- 2, IL- 10, IL- 3 and IL- 5 and GM-CSF, IL- 4 and IL- 13
TLR	4	TLR, TLR/Endogenous ligand, TLR3, Innate immune system	MAPK, ROS/RND/Phagocyte, DAP12, Complement, Fc-epsilon/gamma receptor, C-type lectin receptors, Neutrophil degranulation, Immune system
Fc-epsilon receptor	4	FCeri/Ca, FCeri/MAPK, AT2/NTAL/LAB/Ca, Innate immune system	TLR, ROS/RNS/phagocyte, DAP12, Complement, Fc-gamma receptor, C-type lectin receptors, Neutrophil degranulation, Immune system
TCR	4	Zap70/synapse, CD3/TCR, Second messenger molecules, Adaptive immune system	Immunoregulatory lymphoid/non-lymphoid cells, B cell receptor, MHC Class I antigen, CD28, MHC class II antigen, Immune system

^a^. Hub, High-degree signaling/metabolic pathways in the fibrous cap atheroma plaque network

^b^. Degree, The edges of Hub

^c^. First neighbors, The first level of cellular pathways close to the hub pathway in the fibrous cap atheroma plaque network

^d^. Second neighbors, The second level of cellular pathways close to the hub pathway in the fibrous cap atheroma plaque network

Degree > 3

**Table 8:** Cellular high-degree pathways in the intimal thick/xanthoma plaques

**Hub**^**a**^ (Signaling/Metabolism)	**Degree**^**b**^	**First neighbors**^**c**^ (Signaling/Metabolism)	**Second neighbors**^**d**^ (Signaling/Metabolism)
Signal transduction	6	Receptor tyrosine kinases, Non-receptor tyrosine kinases, WNT, Integrin, GPCR, second messengers	ERBB2, EGFR, ERBB4, PTK6, DAG/IP3/TCF/WNT, Platelet aggregation, GPCR, PIP3/AKT
Netrin- 1	5	DCC, Axon, DSCAM, Netrin, Second messengers/Netrin- 1	Robo receptors, ICAM1
Metabolism	5	Amino acids and derivatives, Abacavir transport, Lipids, Inositol phosphate, vitamins and cofactors	IP2/IP/INs, Abacavir transport, Fatty acid, Amine-derived hormones, Fat-soluble vitamins
EGFR	5	GRB2/EGFR, SHC1/EGFR, GAB1 signalosome, EGFR/Phospholipase C-gamma, Receptor tyrosine kinases	ERBB2, ERBB4, Signal transduction
ERBB2	5	ERBB2/Cell motility, GRB2/ERBB2, PI3 K/ERBB2, ERBB2/PTK6, Receptor tyrosine kinases	EGFR, ERBB4, signal transduction
G alpha	4	Visual phototransduction, PIP2 hydrolysis, GPCR, Opioid	G-protein, GPCR, Platelet activation and aggregation, Retinoid, Retinoid cycle
Receptor tyrosine kinases	4	ERBB2, EGFR, ERBB4, Signal transduction	ERBB2/PTK6, PI3 K/ERBB2, GRB2/ERBB2, ERBB2/cell motility, GRB2/EGFR, SHC1/EGFR, GAB1 signalosome, EGFR/Phospholipase C-gamma, SHC1/ERBB4, PI3 K/ERBB4, WNT, Integrin, Non-receptor tyrosine kinases, GPCR, Second messengers

^a^. Hub, High-degree signaling/metabolic pathways in the intimal thick/xanthoma plaque network

^b^. Degree, The edges of Hub

^c^. First neighbors, The first level of cellular pathways close to the hub pathway in the intimal thick/xanthoma plaque network

^d^. Second neighbors, The second level of cellular pathways close to the hub pathway in the intimal thick/xanthoma plaque network

Degree > 3

The upregulated DEGs were also identified in the stable and unstable plaques as reported in the GSE120521 dataset and were linked with cellular pathways (stable 11, unstable 139). Also, 57 upregulated DEGs were found in 39 pathways by analyzing GSE19590 in atherosclerotic plaques of positive *C. pneumonia* (Cp). The fibrous cap atheroma plaque network was separately enriched using the cellular pathways linked with stable (Fig. 5) and unstable plaques (Fig. 6). Then, the cellular pathways related to the MQ, EC, DC, and VSMC secretory proteins and Cp were enriched into the intimal thick/xanthoma (Fig. 7) and stable/unstable fibrous cap atheroma plaque (Figs. 5 and 6) networks. The intersection nodes between the secretory, stable, and unstable pathways were highlighted on the networks.

## Discussion

It is well known that the atherosclerosis process progresses through cellular dysfunctions and extracellular matrix remodeling in the plaque microenvironment. The activation of cellular pathways affects the movement of monocytes, polarization, proliferation, and migration of vascular cells leading to plaque growth [11]. Pathogens are one of the important agents triggering the pathways in cells [8]. This study provided significant insights into the complex interplay between Gram-negative bacterial infections and, intracellular and intercellular responses for plaque growth. The biology systems analysis of high-throughput expression data revealed how immune and vascular cells exposed to bacterial infections affect plaque stability in the vessel microenvironment.

Many studies have suggested that Gram-negative bacterial infections can exacerbate inflammation [12–14]. Chlamydial infections such as *C. pneumoniae* developed immune events through the INFα/β, TNF-α, MAPK/ERK, NLRP3/ASC/caspase- 1, JAK/STAT, PI3 K/Akt signaling pathways [15]. *P. gingivalis* is abundantly detected in the vessels and is reported to stimulate the production of inflammatory factors in ECs [5]. Furthermore, inflammation elevated by *P. gingivalis* through the NF-κB, c-Jun/AP- 1, JNK, PI3 K/Akt and JAK/Stat PAR2/NF-kB, TLRs, MEK/ERK, Bcl- 2/Bax/caspase- 3 pathways [16, 17]. Triggering these pathways suggested that Gram-negative bacterial infections progress immune responses in MQs, VSMCs, DCs, and ECs. Moreover, the paracrine effects among the activated cells exacerbated the inflammatory events in the plaque microenvironment. Our study confirmed that many of these cellular pathways, including the INFα/β, TNF-α, MAPK, PI3 K, NF-κB, and TLRs, and their cross-talked axes, are activated by Gram-negative bacterial infections in the plaque microenvironment. However, the function and activity of these pathways were different in the cells. In agreement with Xie et al. [18], the study showed that the cytokine, interleukin, TNF, TLR, NF-κB, and INFα/β signaling pathways were activated in the ECs and exacerbated by the MQs. MQs, emerging as central players, not only help to recognize pathogens [19] but also mediate the immune responses through the cytokine, interleukin, GPCR, and TNF signaling pathways. Some studies also reported Chlamydial species trigger cytokine and TNF signaling pathways [14, 20]. Moreover, the study showed that the cellular cross-talks between MQs, VSMCs, DCs, and ECs exacerbate the cytokine, interleukin, GPCR, MAPK, and FLT3 signaling pathways in the MQs.

It is known that the proliferation and migration of VSMCs develop plaque growth [21]. The study showed that Gram-negative bacterial infections activate the NOTCH, GPCR, G alpha, and Interleukin signaling pathways in the VSMCs. In addition to these pathways, the EGFR, Integrin, and TGFβ signaling pathways, involved in the proliferation and migration of VSMCs [22], were also exacerbated by cellular cross-talking with MQs and DCs. The role of dendritic cells was noteworthy considered in Gram-negative bacterial infections [23] triggering the cytokine, Interleukin, GPCR, IFN, FLT3, EGFR, G alpha, Receptor tyrosine kinase, and TLR signaling pathways and exacerbating their activities by the MQs and VSMCs in vessel microenvironment.

The high-throughput expression analysis showed that the GPCR-related signaling pathways are exacerbated by the MQs, VSMCs, DCs, and ECs exposed to Gram-negative bacteria in the intimal thick/xanthoma plaques. Moreover, the innate immune, adaptive immune, receptor tyrosine kinase, cytokine, interleukin, TLR, TCR, and FC-epsilon pathways were activated in the fibrous cap atheroma plaques. These findings also suggested that the active cellular pathways are more observed in the fibrous cap atheroma plaques. Moreover, the GPCR and platelet aggregation pathways and also the GPCR, G alpha, platelet aggregation, cytokine, FLT3, lipid metabolism, and interleukin pathways were exacerbated in the stable and unstable fibrous cap atheroma plaques, respectively.

The results showed that Gram-negative bacterial infections such as *P. gingivalis* and *C. pneumoniae* trigger the different cellular pathways in the ECs, MQs, VSMCs, and DCs. Furthermore, some of these pathways were exacerbated by the pathways linked with the secretory proteins in the vessel microenvironment. The study also highlighted the active cellular pathways in the stable/unstable fibrous cap atheroma and intimal thick/xanthoma plaques. Hub genes and active singling axes in these pathways are suggested as therapeutic targets for managing atherosclerosis [24]. Suppressing these pathways with molecular drugs, molecular constructs containing miRNA/anti-miRNA, antisense oligonucleotides (ASOs), and CRISPR could potentially reduce chronic inflammation and prevent the progression of unstable plaques [25–27].

## Conclusion

The study suggested that Gram-negative bacterial infections trigger and exacerbate the cellular pathways leading to inflammation. Moreover, the findings showed that cellular cross-talking exacerbates the activity of pathways in the plaque microenvironment. However, the study analyzed only the gene expression data. To support the results, we suggested using proteomics data, plaque single-cell data, and other interventional high-throughput data regulating cellular pathways. Further experimental and clinical studies are also needed to investigate these pathways and their potential for therapeutic interventions.

## Supplementary Information

Supplement 1. DEGs in GSEs and E-MATBs.

Supplement 2. Cellular pathways in ECs, MQs, DCs, VSMCs and plaques.

## Data Availability

Data is provided within the manuscript or supplementary information files.

## Abbreviations

Cp: *Chlamydia pneumoniae*
Pg: *Porphyromonas gingivalis*
GEO: Gene expression omnibus
